# Exploring ifosfamide-related central nervous system adverse events based on the FAERS database and clinical feature analysis of cases

**DOI:** 10.3389/fonc.2026.1961478

**Published:** 2026-10-20

**Authors:** Peiqi Shi, Qinghua Mei, Rongjie zheng, Shiqiao Wang

**Affiliations:** Department of Pharmacy, The Affiliated Guangdong Second Provincial General Hospital of Jinan University, Guangzhou, China

**Keywords:** adverse events, central nervous system, FAERS, ifosfamide, pharmacovigilance

## Abstract

**Background:**

Ifosfamide, an alkylating agent used to treat various malignancies, has limited real-world safety data on neurotoxicity. Therefore, this study analyzed ifosfamide-related Central Nervous System Adverse Events (CNS AEs) to provide a reference for drug risk management.

**Methods:**

The disproportionality study was performed leveraging the FDA Adverse Event Reporting System (FAERS) database from 2004 to 2024. Reporting Odds Ratio (ROR), Proportional Reporting Ratio (PRR), Multi-item Gamma Poisson Shrinker (MGPS), and Bayesian Information Component (IC) were applied to identify and evaluate potential CNS AEs associated with ifosfamide. The Weibull distribution was applied to characterize the risk of AEs over time. Case reports from PubMed and SpringerLink were reviewed to summarize the clinical characteristics of these AEs.

**Results:**

This study identified 786 reports of ifosfamide-related CNS AEs. Disproportionality analysis revealed encephalopathy as the strongest signal (n=252, ROR = 101.19). Six label-unlisted adverse events were also identified, including brain oedema, hydrocephalus, spinal cord compression, cerebral ischemia, gaze palsy, and Guillain-Barré syndrome. The time-to-onset analysis revealed an early failure pattern with a median onset of 2 days after drug exposure. Brain oedema exhibited the highest mortality rate (83.33%). Subgroup analysis suggested greater susceptibility to encephalopathy in female patients and to confusion and coma in elderly patients. The systematic case review included 61 cases from 42 articles, involving patients aged 3–77 years. Therein, 46 (75.41%) cases experienced central neurotoxic reactions after receiving one to three cycles of ifosfamide-containing chemotherapy and the common clinical symptoms included impaired consciousness, hallucinations, seizures, delirium, disorientation, and various psychiatric or behavioral abnormalities.

**Conclusions:**

This study corroborates clinical observations by systematically characterizing the features of ifosfamide-related CNS toxicity and identifying potentially label-unlisted AEs, supporting further validation in prospective epidemiological studies.

## 1 Introduction

Ifosfamide, an alkylating agent, has been extensively used against a wide range of cancers since its introduction in 1971 (1). This chemotherapeutic agent can be administered as a monotherapy or in combination with other chemotherapy agents to treat various malignancies, including malignant lymphoma, bone and soft tissue tumors, cervical cancer, ovarian cancer, and breast cancer (2).

Common adverse events (AEs) associated with ifosfamide include nausea, emesis, leukopenia, lymphopenia, and cystitis (3). In particular, ifosfamide may cause various central nervous system (CNS) adverse effects, including decreased alertness, confusion, hallucinations, amnesia, cerebellar dysfunction, muscle weakness, urinary incontinence, and seizures (4). However, the prevalence and severity of these CNS adverse reactions are reported with some variability in clinical studies. Research indicates that 10%-30% of patients experiencing ifosfamide toxicity develop encephalopathy (5, 6). Encephalopathy is more frequently observed in patients with certain predisposing factors, including advanced age, electrolyte imbalances, water intoxication, concomitant renal insufficiency, hepatic dysfunction, low serum albumin levels, bulky or advanced pelvic or retroperitoneal tumor which may cause ureteral obstruction and impaired renal clearance of ifosfamide and its neurotoxic metabolites, poor physical condition, prior central nervous system lesions (such as brain metastases or history of intracranial radiotherapy), and prior cisplatin therapy (7, 8). CNS toxicity associated with ifosfamide can lead to organic brain damage, with the disease progression typically initiating with drowsiness, advancing to coma, and ultimately potentially culminating in death (9). Despite the widespread use of ifosfamide in treating various malignant tumors, its CNS toxicity, combined with the absence of well-established preventive and therapeutic strategies, poses a substantial challenge to its clinical use (10). A retrospective study involving 200 patients with sarcoma and lymphoma identified 29 cases of ifosfamide-associated encephalopathy, primarily characterized by confusion, somnolence, and delirium (11). Among 30 sarcoma patients administered high-dose ifosfamide, two were transitioned to alternative treatments due to ifosfamide-induced encephalopathy (12). Additionally, in a study involving 61 patients with bone and soft tissue sarcomas, one patient died from a seizure during treatment with ifosfamide (13). Current understanding of ifosfamide’s CNS toxicity largely stems from case reports and small-sample retrospective studies, which do not adequately capture its true epidemiological characteristics. Therefore, it is of great significance to conduct a systematic analysis of the clinical manifestations, incidence, time of onset, intervention measures, and prognosis of ifosfamide-related CNS toxicity based on large-scale real-world data.

Based on the above considerations, this study conducted a retrospective analysis that integrated case reports with data from the FAERS database to summarize the clinical characteristics of CNS AEs associated with ifosfamide, with the aim of providing a reference for the clinical identification and management of such adverse reactions.

## 2 Materials and methods

### 2.1 Data source and processing

This study analyzed the clinical characteristics of CNS AEs associated with the Primary Suspected (PS) drug using FAERS reports submitted between Q1 2004 and Q4 2024.

For precise identification, the search included both the generic names “ifosfamide” and “iphosphamide” and the brand names “holoxhan” and “ifex” within the scope of PS drugs. Data cleansing followed the FDA’s FAERS Data Management Manual, where the duplicate reports according to the unique case ID, and the highest primary ID was reserved. The Medical Dictionary for Regulatory Activities version 27.1 (MedDRA 27.1) was employed to identify and extract preferred terms (PTs). A total of 22,249,476 reports were extracted from FAERS, of which 18,627,667 unique cases were retained following deduplication in accordance with FDA guidelines. The detailed screening flowchart is presented in **Figure 1**.

**Figure 1:**
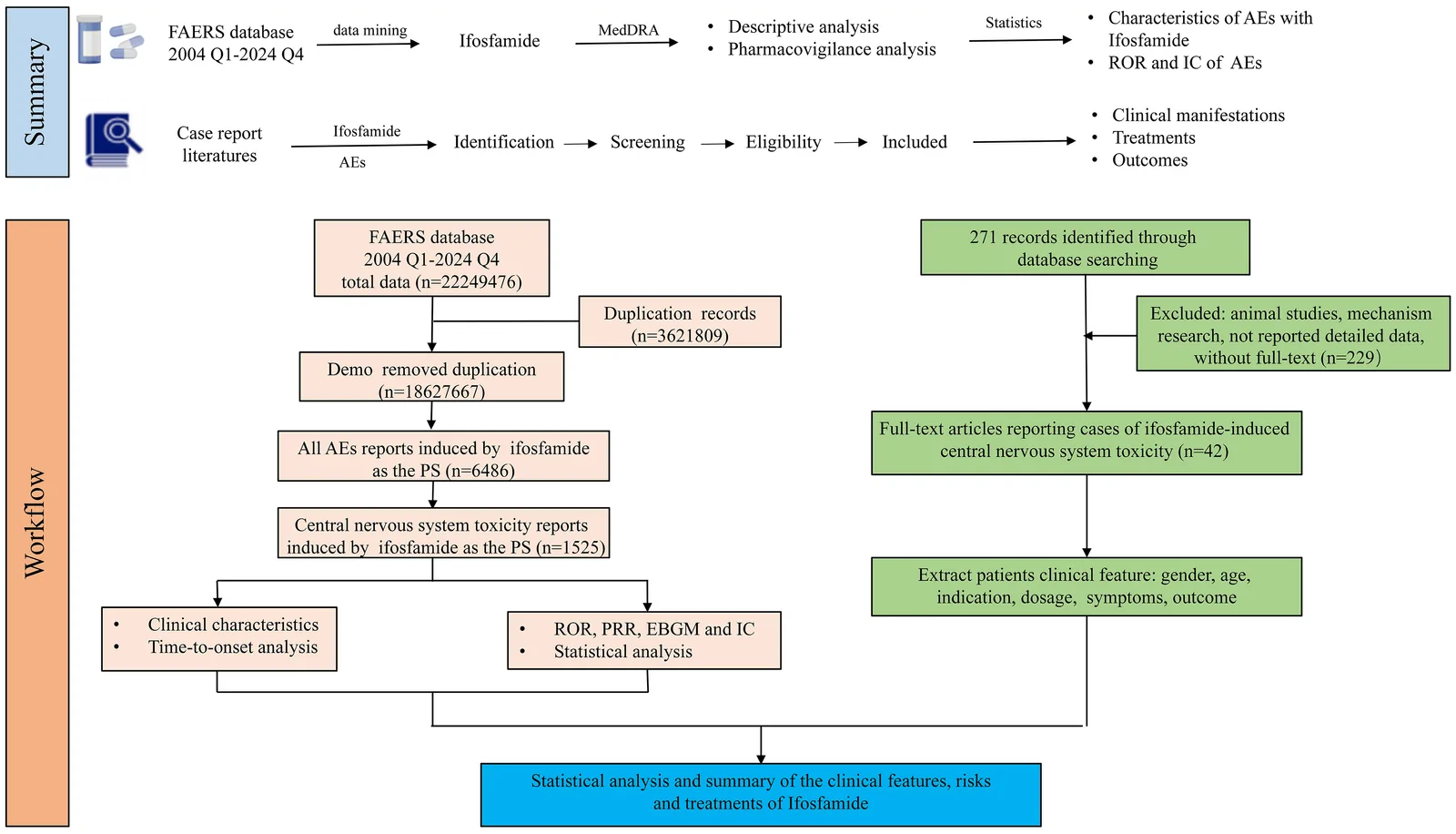
Flowchart of ifosfamide-related adverse events.

### 2.2 Systematic literature review

This study employed search terms comprising the name of the drug ifosfamide and Boolean operators to retrieve case reports and case series related to CNS adverse reactions associated with ifosfamide from several databases, including PubMed, and SpringerLink, covering entries up to September 2025. Two researchers independently screened the literature according to predefined inclusion and exclusion criteria by sequentially reviewing titles, abstracts, and full-text articles, and initially identified 271 relevant articles. Any discrepancies between the two reviewers were resolved through consultation with a third researcher, who determined the final list of included studies. Additionally, a manual retrospective search of the references in the included studies was conducted to identify as many relevant studies as possible.

After preliminary screening, 229 publications were excluded due to animal studies, mechanism research, insufficient data, or inaccessibility of full-text articles. Ultimately, 42 eligible full-text publications were included. For each eligible report, detailed data were systematically extracted, including patient demographics (age and sex), primary disease (indication), ifosfamide dosing regimen, specific neurological manifestations, therapeutic interventions, and clinical outcomes. The methodological quality of the included reports was assessed using an 8-item JBI critical appraisal checklist. Each item was rated as Yes, Unclear, No, or Not Applicable. The number of Yes responses was summed for each study, with a maximum score of 8. The proportion of studies fulfilling each JBI item was also calculated. Two reviewers independently conducted the quality assessment, and discrepancies were resolved by discussion or arbitration by a third reviewer. Because the included studies were predominantly case reports and case series, the findings were synthesized descriptively. Categorical variables were summarized as frequencies and percentages. The mean JBI Yes score and the distribution of studies according to total Yes score were calculated.

### 2.3 Data analysis

This study used disproportionality analysis to identify safety signals for drugs. Four disproportionality analysis methods were used to identify signals of potential adverse reactions associated with ifosfamide: Reporting Odds Ratio (ROR), Proportional Reporting Ratio (PRR), Multi-item Gamma Poisson Shrinker (MGPS), and Bayesian Information Component (IC) (14). To ensure signal accuracy and mitigate false positives, AEs signals were deemed significant only when meeting the criteria of all four algorithms simultaneously (15). Detailed two-by-two contingency tables are provided in **Supplementary Table S1**. Specific formulas and identification thresholds for each algorithm are provided in **Supplementary Table S2**.

Additionally, we employed descriptive analysis to summarize the demographic characteristics of the reports, including gender, age, reporting country, reporter, and outcome. Moreover, the temporal relationship between ifosfamide initiation and AEs onset was quantified as Time-To-Onset (TTO), derived from the interval between EVENT_DT (AEs onset date, DEMO file) and START_DT (therapy initiation date, THER file). The Weibull distribution was applied to characterize temporal evolution of AEs incidence. All data processing and statistical analysis were conducted using R software (version 4.5.0) and Microsoft Excel 2021.

## 3 Results

### 3.1 Basic characteristics of AEs

During the period from Q1 2004 to Q4 2024, the FAERS database recorded 1,525 reports of central nervous system AEs. Specific details were presented in **Table 1**. Ifosfamide-related AEs were more frequently reported in females (n=354, 47.33%) than in male (n=297, 37.79%). Adult patients (n=354, 45.04%) receiving ifosfamide were significantly more frequently reported CNS AEs compared to other age groups. The distribution of reports by body weight was as follows: <50 kg (n=64, 8.14%), 50–100 kg(n=171, 21.76%), and >100 kg(n=22, 2.80%). The 93 deaths accounted for 11.83% of the serious AEs. Most of AEs were reported from the United States (44.27%). More than half (51.15%) of the reports were submitted by healthcare professionals.

**Table 1:** Characteristics of patients with ifosfamide-related CNS toxicity.

Characteristics	Ifosfamide associated central nervous system toxicity (n=786)		Ifosfamide associated overall ADE (n=2385)	
	Available number	Value	Available number	Value
Gender, n (%)	669 (85.11%)	–	1837 (77.02%)	–
Female	–	372 (47.33%)	–	908 (38.07%)
Male	–	297 (37.79%)	–	929 (38.95%)
Age (years), n (%)	600 (76.34%)	–	1664 (69.77%)	–
<18	–	131 (16.67%)	–	438 (18.36%)
18≤and ≤ 64	–	354 (45.04%)	–	997 (41.8%)
>64	–	115 (14.63%)	–	229 (9.6%)
Weight (kg), n (%)	257 (32.7%)	–	754 (31.61%)	–
<50 kg	–	64 (8.14%)	–	213 (8.93%)
50∼100 kg	–	171 (21.76%)	–	481 (20.17%)
>100 kg	–	22 (2.8%)	–	60 (2.52%)
Serious outcome, n (%)	786 (100%)	–	2385 (100%)	–
Death	–	93 (11.83%)	–	478 (20.04%)
Life-threatening	–	62 (7.89%)	–	135 (5.66%)
Hospitalization	–	269 (34.22%)	–	760 (31.87%)
Disability	–	7 (0.89%)	–	19 (0.8%)
Others serious outcomes	–	355 (45.17%)	–	993 (41.64%)
Reported Countries, n (%)	786 (100%)	–		–
US	–	348 (44.27%)	–	829 (34.76%)
Non-US	–	438 (55.73%)	–	1556 (65.24%)
Reported Person, n (%)	491 (62.47%)	–	1474 (61.8%)	–
Physician	–	181 (23.03%)	–	824 (34.55%)
Pharmacist	–	169 (21.5%)	–	251 (10.52%)
Other health-professional	–	52 (6.62%)	–	179 (7.51%)
Consumer	–	89 (11.32%)	–	220 (9.22%)

### 3.2 Disproportionality analysis of central neurological toxicity

A total of 42 PTs CNS AEs related to ifosfamide were identified in the FAERS database. The most frequently reported PTs were encephalopathy (n=252), confusional state (n=120), and neurotoxicity (n=117). The disproportionality analysis revealed statistically significant signal strengths for all 42 PTs, with ROR values ranging from 3.19 (95% CI: 2.06-4.95) for lethargy to 101.19 (95% CI: 89.16–114.85) for encephalopathy. The strongest signals were observed for encephalopathy (ROR = 101.19), neurotoxicity (ROR = 67.42), and toxic encephalopathy (ROR = 66.17). Notably, six potential safety signals were identified for AEs not currently listed on the ifosfamide drug label, including brain oedema, hydrocephalus, spinal cord compression, cerebral ischemia, gaze palsy, and Guillain-Barré syndrome. These signals, identified through disproportionality analysis of spontaneous reports, warrant further clinical investigation to determine whether the observed reporting associations reflect causal relationships. Detailed information is presented in **Table 2**.

**Table 2:** Signal strength of CNS toxicity reports of ifosfamide at the PT level in FAERS database.

Preferred term	n	Reporting odds ratio (95%)	Proportional reporting ratio (χ^2^)	Empirical Bayes geometric mean (EBGM05)	Information component (IC025)
Encephalopathy	252	101.19 (89.16 - 114.85)	97.3 (23751.41)	96.19 (86.52)	6.59 (6.4)
Confusional state	120	6.92 (5.78 - 8.29)	6.81 (596.32)	6.81 (5.85)	2.77 (2.5)
Neurotoxicity	117	67.42 (56.11 - 81)	66.22 (7458.32)	65.71 (56.35)	6.04 (5.77)
Disorientation	57	13.1 (10.09 - 17.01)	13 (630.74)	12.98 (10.43)	3.7 (3.32)
Agitation	40	4.96 (3.63 - 6.77)	4.94 (125.62)	4.93 (3.8)	2.3 (1.85)
Mental status changes	39	13.01 (9.49 - 17.83)	12.94 (429.15)	12.92 (9.93)	3.69 (3.23)
Toxic encephalopathy	33	66.17 (46.94 - 93.29)	65.84 (2090.98)	65.34 (49.02)	6.03 (5.53)
Hallucination	28	3.52 (2.43 - 5.11)	3.51 (50.34)	3.51 (2.57)	1.81 (1.27)
Myoclonus	25	19.55 (13.19 - 28.97)	19.48 (437.35)	19.44 (13.99)	4.28 (3.71)
Coma	25	4.84 (3.27 - 7.17)	4.83 (75.82)	4.82 (3.47)	2.27 (1.7)
Lethargy	20	3.19 (2.06 - 4.95)	3.19 (30.02)	3.19 (2.21)	1.67 (1.04)
Dyskinesia	17	3.85 (2.39 - 6.2)	3.84 (35.79)	3.84 (2.58)	1.94 (1.26)
Delirium	15	4.1 (2.47 - 6.81)	4.1 (35.12)	4.1 (2.68)	2.03 (1.31)
Status epilepticus	15	12.5 (7.53 - 20.75)	12.47 (158.06)	12.45 (8.15)	3.64 (2.92)
Aphasia	14	4.23 (2.5 - 7.15)	4.22 (34.43)	4.22 (2.72)	2.08 (1.33)
Posterior reversible encephalopathy syndrome	14	14.26 (8.43 - 24.1)	14.23 (171.91)	14.21 (9.16)	3.83 (3.08)
Depressed level of consciousness	14	3.25 (1.93 - 5.5)	3.25 (21.79)	3.25 (2.09)	1.7 (0.95)
Unresponsive to stimuli	13	4.74 (2.75 - 8.17)	4.73 (38.29)	4.73 (3)	2.24 (1.47)
Altered state of consciousness	11	4.89 (2.71 - 8.84)	4.89 (33.98)	4.88 (2.98)	2.29 (1.45)
Hallucination, visual	10	4.56 (2.45 - 8.49)	4.56 (27.78)	4.56 (2.71)	2.19 (1.32)
Incoherent	8	11.12 (5.56 - 22.25)	11.11 (73.48)	11.09 (6.21)	3.47 (2.51)
Metabolic encephalopathy	8	24.7 (12.34 - 49.47)	24.67 (181.18)	24.6 (13.76)	4.62 (3.66)
Neurological decompensation	7	29.17 (13.88 - 61.3)	29.14 (189.58)	29.04 (15.6)	4.86 (3.84)
Sopor	7	4.61 (2.19 - 9.67)	4.6 (19.73)	4.6 (2.47)	2.2 (1.18)
Coordination abnormal	7	4.48 (2.13 - 9.39)	4.47 (18.86)	4.47 (2.4)	2.16 (1.14)
Psychomotor hyperactivity	7	3.78 (1.8 - 7.94)	3.78 (14.31)	3.78 (2.03)	1.92 (0.9)
Apraxia	6	37.99 (17.03 - 84.76)	37.96 (214.95)	37.79 (19.31)	5.24 (4.15)
Brain oedema*	6	4.43 (1.99 - 9.87)	4.43 (15.92)	4.43 (2.27)	2.15 (1.05)
Hydrocephalus*	5	8.45 (3.52 - 20.33)	8.45 (32.8)	8.44 (4.05)	3.08 (1.9)
Psychomotor skills impaired	5	17.15 (7.13 - 41.26)	17.14 (75.84)	17.11 (8.21)	4.1 (2.92)
Peripheral sensory neuropathy	5	8.2 (3.41 - 19.72)	8.2 (31.57)	8.19 (3.93)	3.03 (1.85)
Hypotonia	5	4.55 (1.89 - 10.94)	4.55 (13.83)	4.55 (2.18)	2.18 (1.01)
Leukoencephalopathy	5	12.13 (5.05 - 29.18)	12.13 (50.97)	12.11 (5.81)	3.6 (2.42)
Spinal cord compression*	4	9.3 (3.49 - 24.8)	9.3 (29.58)	9.29 (4.09)	3.22 (1.92)
Cerebral ischemia*	4	6.82 (2.56 - 18.18)	6.81 (19.83)	6.81 (3)	2.77 (1.48)
Gaze palsy*	3	16.45 (5.3 - 51.08)	16.44 (43.43)	16.41 (6.36)	4.04 (2.59)
Tonic clonic movements	3	21.44 (6.9 - 66.58)	21.43 (58.27)	21.38 (8.28)	4.42 (2.97)
Guillain-Barré syndrome*	3	5.94 (1.91 - 18.42)	5.94 (12.31)	5.93 (2.3)	2.57 (1.12)
Petit mal epilepsy	3	5.53 (1.78 - 17.15)	5.53 (11.11)	5.52 (2.14)	2.47 (1.02)
Behaviour disorder	3	5.96 (1.92 - 18.48)	5.95 (12.36)	5.95 (2.31)	2.57 (1.13)
Clumsiness	3	9.87 (3.18 - 30.65)	9.87 (23.89)	9.86 (3.82)	3.3 (1.86)
Cerebellar syndrome	3	12.35 (3.98 - 38.33)	12.34 (31.22)	12.33 (4.78)	3.62 (2.18)

*Not mentioned in the ifosfamide instruction.

### 3.3 Sensitivity analysis

Ifosfamide is frequently administered in combination with agents such as cisplatin, carboplatin, epirubicin, etoposide, and mesna. To rule out the potential impact of other concomitant medications, this study excluded AEs related to these aforementioned concomitant medications, and the results show that AEs are significantly reduced. However, signals such as encephalopathy, confusional state, neurotoxicity, disorientation, agitation, mental status changes, toxic encephalopathy and coma remain stable (**Supplementary Table S3**).

### 3.4 Analysis of time to onset and weibull distribution

Researchers collected data on the time to event onset of ifosfamide-related AEs from the database. A total of 233 cases had clearly documented time-to-onset data, accounting for 29.64% of the total 786 cases. The median time to event onset was 2 days (interquartile range [IQR]: 1–8 days). The time-to-onset distribution was presented in **Figure 2**. Among the 233 cases with documented time-to-onset data, 204 (87.55%) reported ifosfamide-related AEs within 30 days. A further 13 (5.58%), 10 (4.29%), and 6 (2.58%) cases reported AEs between 31 and 60 days, 61 and 90 days, and 91 days to 1 year after treatment initiation, respectively. It is noteworthy that a small proportion (2.58%) of AEs may still be reported between 3 months and up to one year after treatment. The Weibull shape parameter (β) characterizes the temporal change in the hazard of AE onset. The estimated β was 0.62 (95% CI: 0.57-0.68), with the entire confidence interval below 1, indicating an early-failure pattern. In simple terms, this pattern suggests that AEs in the analyzed reports tended to have earlier documented onset after treatment initiation rather than later. The detailed parameters are listed in **Table 3**.

**Figure 2:**
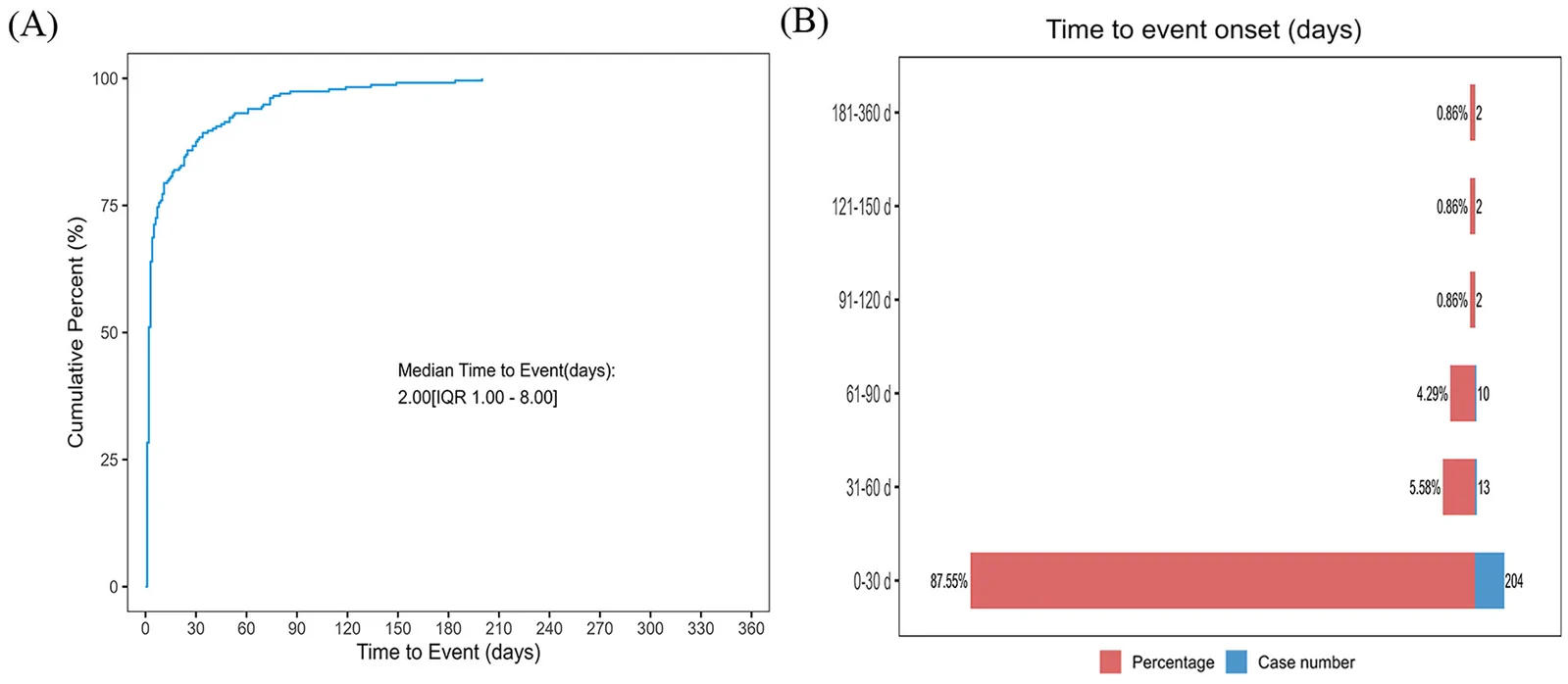
**(A)** Cumulative curve of adverse reaction induction time, **(B)** Time to event onset of ifosfamide-related AEs.

**Table 3:** Weibull shape parameter test for CNS associated with ifosfamide.

Drug	Cases	TTO (days)		Weibull distribution				Failure type
	n	Median (IQR)	Min–Max	Scale parameter		Shape parameter		
				α	95% CI	β	95% CI	
ifosfamide	233	2 (1–8)	1-200	8.12	6.42-10.00	0.62	0.57-0.68	Early failure

n, number of cases with available time-to-onset; IQR, interquartile range; TTO, Time-to-onset.

### 3.5 Analysis of death outcome

This study further evaluated the association between various CNS toxicities and fatal outcomes. Among these AEs, brain oedema had the highest proportion of reported fatal outcomes at 83.33%. The next highest proportions of reported fatal outcomes were observed for neurological decompensation (57.14%, 4/7) and coma (56.00%, 14/25). Although cases of spinal cord compression (50.00%, 2/4) were relatively few, they showed a similarly high proportion of reported fatal outcomes. In contrast, although the proportions of reported fatal outcomes for confusion (5.83%, 7/120), disorientation (8.77%, 5/57), systemic neurotoxicity (9.40%, 11/117), and encephalopathy (11.11%, 28/252) exhibited relatively lower mortality rates. However, due to their higher reporting frequencies at baseline, these symptoms still accounted for a significant proportion of total fatal outcome. Detailed information is presented in **Figure 3**.

**Figure 3:**
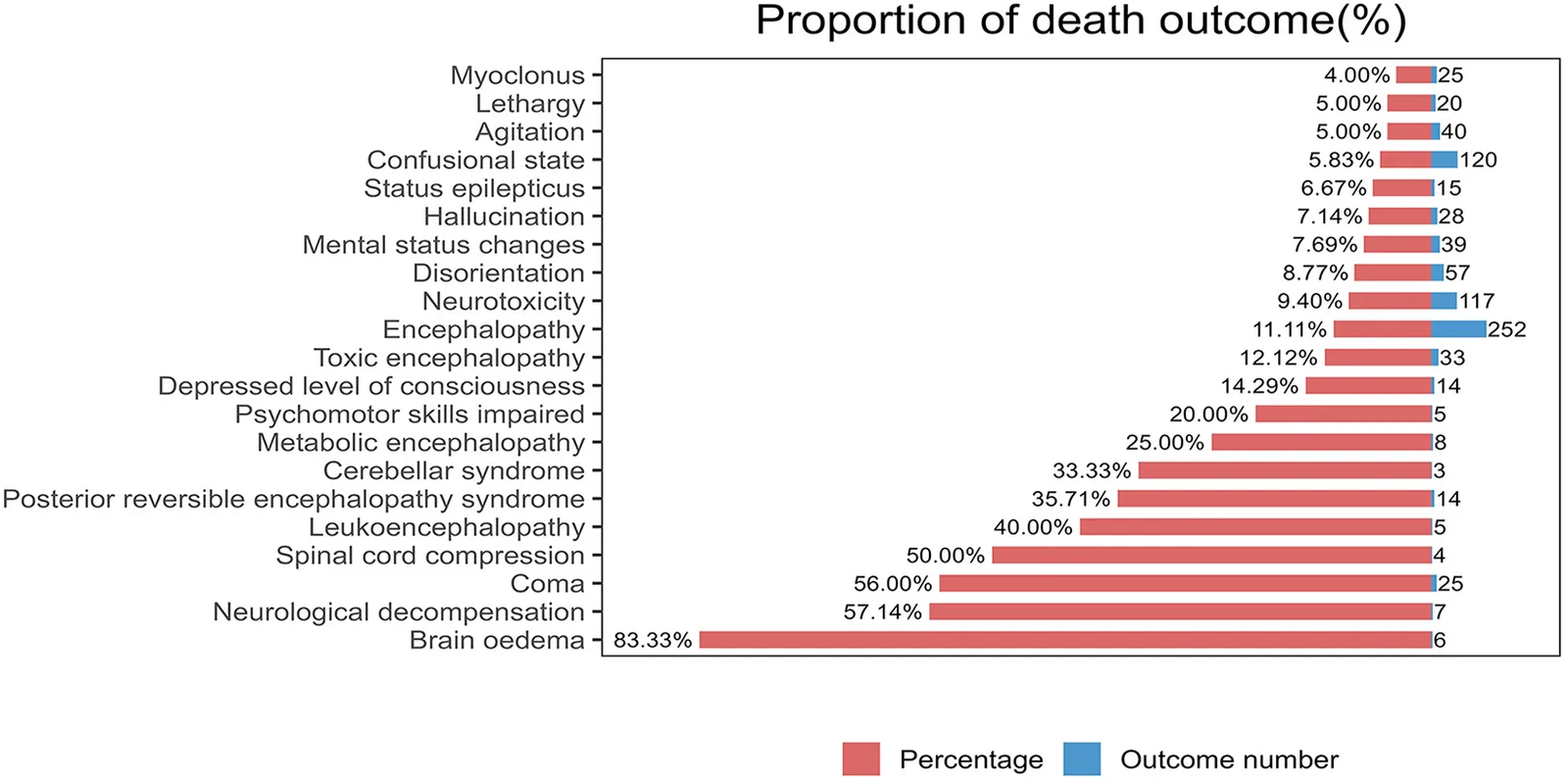
The proportion of death outcome related to ifosfamide.

### 3.6 Analysis of gender and age subgroups

Analysis of gender and age subgroups was conducted in this study. As shown in **Figure 4A**, signal detection identified 27 PTs in the female cohort, exceeding the 23 PTs identified in the male cohort. Encephalopathy was the most frequently reported event and exhibited the highest ROR. 120 cases of encephalopathy were reported in female patients with ROR of 135.64 (95% CI: 112.87-163.02), significantly higher than the 65 cases and ROR of 47.08 (95% CI: 36.78-60.26) observed in males. ROR values for other common events such as confusional state, neurotoxicity, and disorientation are typically higher in females than in males.

**Figure 4:**
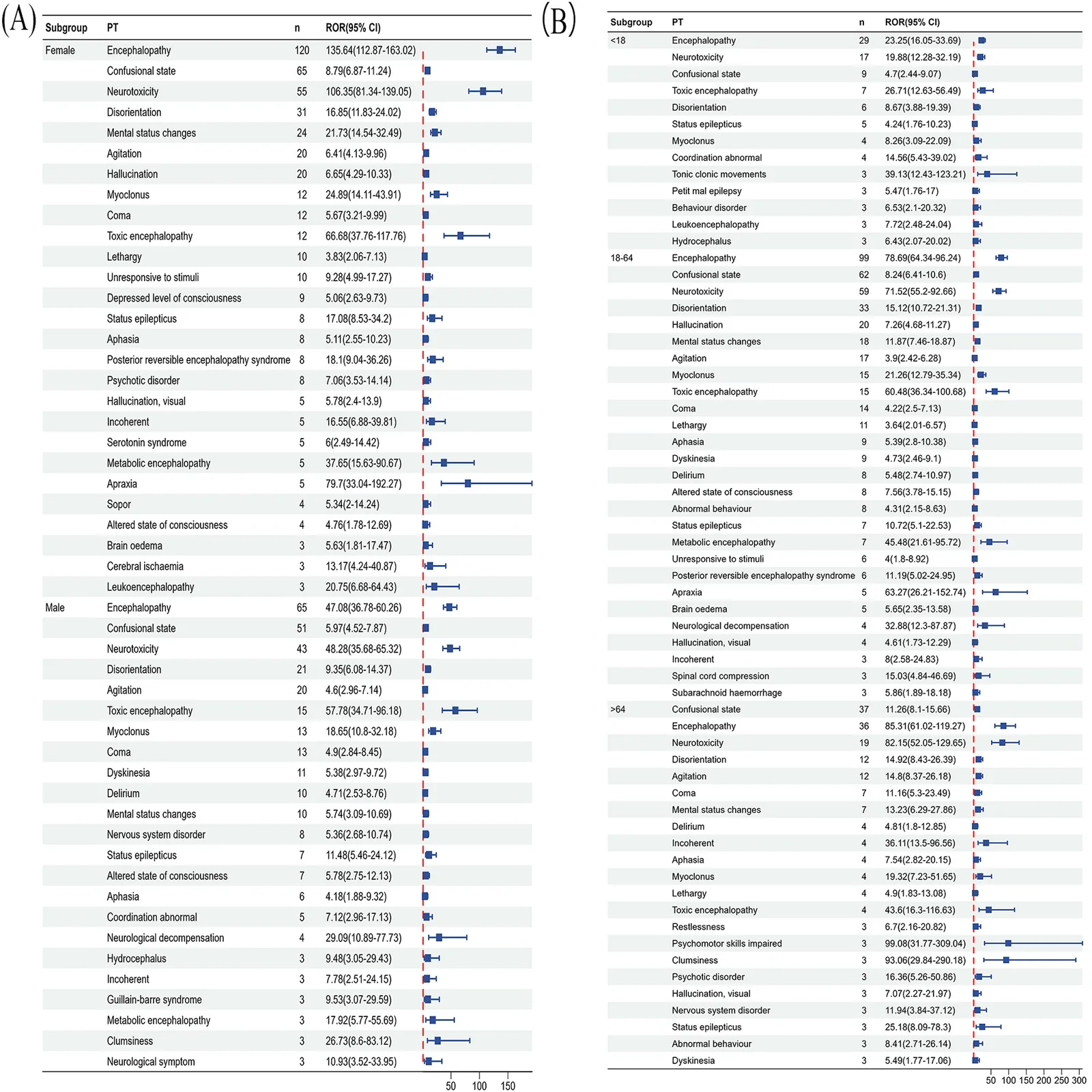
Distribution of central neurological AEs to ifosfamide in different subgroups. **(A)** Gender; **(B)** Age.

Age-stratified analysis showed that the minors subgroup (<18 years) exhibited reported events such as tonic clonic movements, petit mal epilepsy, and behavioral disorders that were not observed in other age groups (**Figure 4B**). Although encephalopathy and neurotoxicity had the highest reporting rates, their ROR values remained relatively low. The adult group (18–64 years) exhibited the widest spectrum of adverse events (27), with ifosfamide-related encephalopathy showing the strongest signal (ROR = 78.69). In the elderly subgroup (>64 years), confusional state was the most frequently reported event, while psychomotor impairment (ROR = 99.08), clumsiness (ROR = 93.06), and encephalopathy (ROR = 85.31) demonstrated very strong signals.

### 3.7 Review of the cases

The methodological quality of the included studies is summarized in **Supplementary Table S5**. Overall, the included case reports and case series were of moderate-to-high methodological quality: the mean number of items rated “yes” was 7.9/8, and 38/42 (90.5%) studies satisfied all eight items. A total of 61 cases of ifosfamide-related central neurotoxicity were identified from 42 published reports (**Supplementary Table S4**). The patients ranged in age from 3 to 77 years with adults accounting for the majority (n=39, 63.93%), and females represented a higher proportion of cases (n=37, 60.66%). The primary clinical indications for ifosfamide treatment included sarcoma, lymphoma, ovarian cancer, breast cancer, and other solid tumors. Most patients (n=46, 75.41%) experienced central neurotoxic reactions after receiving one to three cycles of ifosfamide-containing chemotherapy, while a few cases developed severe central nervous system symptoms after four cycles. The common clinical symptoms included impaired consciousness, hallucinations, seizures, delirium, disorientation, and various psychiatric or behavioral abnormalities. Overall, 29/46 (63.04%) recovered within 48 h and 38/46 (82.61%) within 72 h, with reported recovery times ranging from several minutes to 3 weeks. Recovery was reported in 54/61 patients (88.52%), whereas 7/61 had a fatal outcome. The most frequently reported treatments were methylene blue (47.54%), benzodiazepines (26.23%), antipsychotics (18.03%), and thiamine (13.11%), with several other agents used less frequently, often as combination therapy. Detailed summary information on the reported treatments is provided in **Supplementary Table S6**.

## 4 Discussion

Although ifosfamide is widely used in clinical practice, previous studies have shown that it may induce CNS toxicity, which can lead to treatment interruption or even pose life-threatening risks to patients (16). Therefore, this research performed an in-depth evaluation of ifosfamide-related central neurotoxic AEs through a pharmacovigilance assessment and a systematic review of case reports.

This study revealed the diversity and severity of CNS toxicity associated with ifosfamide. Disproportionality analysis identified the most commonly reported and significant signals for AEs such as encephalopathy (n=252, ROR = 101.19) and neurotoxicity (n=117, ROR = 67.42). Currently, the CNS toxicity associated with ifosfamide therapy has not yet been fully understood. Recent studies have also identified chloroacetaldehyde, an ifosfamide metabolite, as a potential cause of central nervous system toxicity (17). The depletion of glutathione induced by chloroacetaldehyde may exacerbate systemic toxicity, particularly neurotoxicity (18). Chloroacetaldehyde may exert neurotoxic effects through direct neurotoxic mechanisms or by impairing fatty acid metabolism via inhibition of mitochondrial oxidative phosphorylation (19). The frequent reporting of confusional state with low signal intensity indicates that although certain clinically common adverse events are frequently reported, this may be due to the concomitant use of other drugs with similar risks or disease progression (20, 21). Of particular note, this study identified six neurological AEs not listed in the drug’s prescribing information, including brain oedema, hydrocephalus, spinal cord compression, cerebral ischemia, gaze palsy, guillain-barre syndrome. Although these signals were identified through the disproportionality analysis, further clinical investigation is required to validate their causal relationship, they lack specificity and are often misinterpreted as indicative of tumor progression or accompanying drug toxicity, which may lead to delayed diagnosis. For example, methotrexate and cytarabine may cause central neurotoxicity, including encephalopathy, leukoencephalopathy, or cerebellar dysfunction. Fluoropyrimidines, particularly 5-fluorouracil, have also been associated with acute encephalopathy, including hyperammonemia encephalopathy in susceptible patients, and high-dose etoposide has been reported in association with acute encephalopathy (22, 23). In addition, cerebral edema and hydrocephalus may be misdiagnosed as intracranial metastases, spinal cord compression may initially be attributed to vertebral metastasis, and Guillain-Barré syndrome to chemotherapy-related fatigue or tumor-associated neuropathy (24–27). These diagnostic challenges highlight the need for clinical vigilance, while the identified signals should be regarded as potential reporting associations requiring further clinical and pharmacoepidemiological validation.

Our findings indicate that the onset of this symptom is unusually rapid, with a median onset time of 2 days post-administration (IQR: 1–8 days). This acute onset pattern likely stems primarily from the rapid accumulation of its neurotoxic metabolite, chloroacetaldehyde, leading to immediate disruption of brain mitochondrial function and neurotransmitter homeostasis (28). Delayed metabolite clearance is a key factor increasing the risk of neurotoxicity, which may stem from congenital enzyme deficiency or result from hepatic or renal failure (29). Previous use of cisplatin has been shown to heighten this risk due to reduced renal clearance (30). The data indicate that the first treatment cycle represents a high-risk window for central neurotoxicity, particularly within the first 48 hours after drug infusion begins (31, 32). The Weibull model further reveals that the risk of CNS toxicity associated with ifosfamide decreases over time. During the high-risk period, clinicians should closely monitor for any changes in their mental state, emotional disturbances, hallucinations, or impaired motor coordination, in order to take the necessary intervention measures in a timely manner. The AEs observed months after treatment initiation are relatively rare. It is essential to remain vigilant throughout the entire treatment process, continuously monitoring patients for adverse reactions, particularly in those receiving long-term medication.

Different types of CNS toxicity reactions exhibit significant variations in lethal risk. Significantly, our analysis of mortality related to CNS AEs during ifosfamide treatment identified brain oedema as having the highest proportion of reported fatal outcomes at 83.33%. The finding is consistent with previous evidence suggesting that brain oedema represents the most common final pathway to brain death (33). The cellular mechanisms of brain oedema are categorized into three distinct types: cytotoxic, vascular, and hydrostatic oedema (34). The severity of ifosfamide-related central neurotoxicity is determined not only by the drug itself, but also significantly by the patient’s underlying condition and prior treatment history. D J Husband et al. reported a case of a patient who developed severe hypokalemia following ifosfamide chemotherapy and ultimately died (35). Research indicates that the development of severe neurological complications, such as fatal encephalopathy, during the treatment of pediatric brain tumors with ifosfamide may be associated with tumor-induced disruption of the blood-brain barrier and radiation therapy (36). Therefore, it is recommended that patients undergo a comprehensive assessment of their underlying conditions and metabolic capacity prior to receiving ifosfamide therapy to prevent serious adverse events such as death.

Patients of different genders have varying physiological conditions, and therefore respond differently to medications. In this study, among patients with available sex data who had documented CNS AEs, females outnumbered males. This gender difference may be attributed to ifosfamide requiring activation by hepatic cytochrome P450 enzymes to exert its anticancer effects and generate toxic metabolites (37). Research indicates that women may exhibit higher CYP3A4 activity than men, potentially leading to faster conversion of ifosfamide into neurotoxic metabolites (38). Additionally, women’s higher body fat percentage may influence drug distribution and accumulation within the body, collectively contributing to increased neurotoxicity risk (39).

This study also found that the ifosfamide-related AEs were more frequently reported in patients aged 18–64 years compared to other age groups, which may be related to the specific characteristics of its therapeutic indications for treating tumors. Neurotoxicity was more frequently reported in adults, likely because this age group constitutes the primary patient cohort for sarcomas, lymphomas, and other cancers, which are typically treated with high-dose regimens containing this medication (40). In contrast, fewer cases are reported among elderly and minors. However, their potential risk should not be overlooked, as age-related pharmacokinetic changes may exacerbate toxicity. The CNS toxicity profile induced by ifosfamide in minors primarily includes altered mental status, cerebellar dysfunction, and seizures. The minors reported distinct neurotoxicities such as tonic clonic movements and coordination abnormalities. The occurrence of ifosfamide-related encephalopathy in children is unaffected by infusion methods or dosage, and is unrelated to known intracranial risk factors, suggesting a potentially distinct pathogenesis (41). Retrospective studies confirm that advanced age is an independent risk factor for ifosfamide-related encephalopathy (42). This may be related to the accumulation of toxic drug metabolites in the body due to renal decline and changes in body composition in the elderly (43). Early reports attributed transient confusion in adults following ifosfamide administration to advanced age, but subsequent studies indicated that these CNS symptoms are not solely determined by age factors, but primarily occur with high-dose bolus administration or 24-hour continuous intravenous infusion of ifosfamide (44).

The aggregated case reports are consistent with previous research results, showing that ifosfamide-related central neurotoxicity occurs more frequently in female patients than in males, and is characterized by a diverse range of clinical symptoms (37). Such events most commonly arise during the first treatment cycle but may also occur in subsequent cycles. As a potentially life-threatening adverse reaction, it warrants a high level of clinical vigilance. In terms of management, prompt discontinuation of ifosfamide remains the primary and most critical measure for controlling central neurotoxicity. Methylene blue may function as an alternative electron acceptor, substituting for inhibited flavoproteins and thereby restoring mitochondrial respiratory chain activity (45). It can also oxidize NADH (Nicotinamide adenine dinucleotide) to facilitate aldehyde dehydrogenase-mediated reactions, and may inhibit plasma and extrahepatic monoamine oxidases (46). Therefore, for patients presenting with severe symptoms such as hallucinations or impaired consciousness, early administration of methylene blue could be considered a therapeutic strategy.

Attribution of CNS adverse events should be interpreted in the context of tumour-specific treatment settings and competing diagnoses. In sarcoma, ifosfamide may be administered in dose-intensive or multi-cycle regimens, and cumulative exposure, infusion schedule, prior cisplatin treatment, renal function, and hypoalbuminemia may influence the likelihood and presentation of encephalopathy (47). In lymphoma, neurological symptoms may occur in the setting of multi-agent chemotherapy, CNS infection, metabolic disturbances, or CNS involvement by the underlying disease (40, 48). In pelvic or gynecological malignancies, bulky pelvic disease or urinary obstruction may impair renal clearance and complicate the interpretation of neurological symptoms (3, 49). In the present literature review, breast cancer accounted for only 3 of 61 independent patients, consistent with the limited use of ifosfamide in breast cancer. In breast cancer, neurological symptoms during ifosfamide therapy should be distinguished from CNS metastases, cerebrovascular or metabolic events, and other treatment-related toxicities (50, 51). A recent dual-database pharmacovigilance study identified potential seizure reporting signals for lapatinib, tucatinib, trastuzumab, trastuzumab emtansine, and atezolizumab during breast cancer treatment (52). These findings do not validate an ifosfamide signal or establish causality, but they reinforce the need to assess the complete treatment history and alternative etiologies before attributing a neurological event to ifosfamide.

The median reported time to onset of neurotoxicity was 2 days, supporting intensified neurological monitoring early during ifosfamide therapy. Enhanced surveillance is warranted in female and older patients who showed differential reporting patterns in our subgroup analysis and in those with renal or hepatic dysfunction, hypoalbuminemia, electrolyte abnormalities, prior ifosfamide or cisplatin exposure, short-duration infusions, bulky pelvic disease, CNS involvement, or potentially interacting co-medications, particularly during the first treatment course. Before each treatment course, baseline mental status should be documented, and renal function, serum albumin, electrolytes, hepatic function, concomitant medications, and relevant disease-related factors should be reviewed. During treatment, clinicians should monitor for new confusion, somnolence, disorientation, agitation, hallucinations, speech disturbance, tremor, myoclonus, seizures, or reduced consciousness. Any new mental status change should prompt immediate interruption of ifosfamide, clinical assessment, and evaluation for reversible or alternative causes. Patients presenting with coma, suspected neurological decompensation, cerebral oedema, or spinal cord compression require urgent neuroimaging and specialist assessment. Supportive care and correction of reversible abnormalities remain the mainstays of management. Methylene blue or thiamine may be considered under institutional protocols and specialist judgement.

## 5 Study limitations

This study has several limitations. First, FAERS is a spontaneous reporting system, and issues such as incomplete reporting, duplicate entries, incomplete case information, and missing data on underlying conditions and concomitant medications may lead to data inaccuracies (53). Important confounders, including renal function, albumin and electrolyte levels, prior cranial radiotherapy, tumor progression, comorbidities, treatment regimen, and complete concomitant medication history, were not systematically available in FAERS. Therefore, despite the sensitivity analysis excluding selected concomitant medications, we could not fully exclude residual confounding. Second, the disproportionality analysis only reflects a statistical association between the drug and AEs and cannot directly prove causality (54). The observed signals may be influenced by underlying disease, tumor progression, co-medications, or reporting practices. Finally, although a systematic literature review was incorporated to complement real-world data, the available evidence is predominantly derived from case reports and small case series and is therefore susceptible to publication bias. In addition, as the JBI appraisal indicated, a minority of reports did not fully describe diagnostic work-up or intervention details, which further limits the precision of the pooled estimates. Therefore, further research is needed to elucidate the demographic characteristics, risk stratification, and effective prevention strategies for ifosfamide-induced central nervous system toxicity.

## 6 Conclusion

This study undertook a comprehensive analysis of ifosfamide-related CNS AEs occurring during ifosfamide treatment, identifying multiple disproportionality signals including encephalopathy, neurotoxicity, and toxic encephalopathy. Some AEs not yet mentioned in the current drug label include brain oedema, hydrocephalus, spinal cord compression, cerebral ischemia, gaze palsy, and Guillain-Barré syndrome. The findings provide more reliable safety evidence for clinical practice, aiding in the development of individualized treatment strategies and strengthening medication risk management. Furthermore, these findings can assist clinicians in the early recognition of ifosfamide-related neurological AEs, providing a reference for timely drug intervention.

## Data Availability

The datasets presented in this study can be found in online repositories. The names of the repository/repositories and accession number(s) can be found in the article/**Supplementary Material**.
