# Supplementary material for: Exploring ifosfamide-related central nervous system adverse events based on the FAERS database and clinical feature analysis of cases
Source: Front Oncol. 2026 Oct 20;16:1961478. doi: 10.3389/fonc.2026.1961478 (PMC13727380; doi:10.3389/fonc.2026.1961478)
Supplement: Supplementary file 1 [file Table1.docx]

**Supplementary Table S1** Two-by-two contingency table for disproportionality analyses

|  | **Target AE** | **Other AE** | **Total** |
| --- | --- | --- | --- |
| Target drug | a | b | a+b |
| Other drugs | c | d | c+d |
| Total | a+c | b+d | a+b+c+d |

**Supplementary Table S2** Summary of four major algorithms used for signal detection

| Algorithms | Equation | Criteria |
| --- | --- | --- |
| ROR | ROR=ad/b/c | lower limit of 95% CI>1, N≥3 |
|  | 95%CI=e^ln(ROR)±1.96(1/a+1/b+1/c+1/d)^0.5^ |  |
| PRR | PRR=a(c+d)/c/(a+b) | PRR≥2, χ^2^≥4, N≥3 |
|  | χ^2^=[(ad-bc)^2](a+b+c+d)/[(a+b)(c+d)(a+c)(b+d)] |  |
| BCPNN | IC=log_2_a(a+b+c+d)/(a+c)(a+b) | IC025>0 |
|  | 95%CI= E(IC) ± 2V(IC)^0.5 |  |
| MGPS | EBGM=a(a+b+c+d)/(a+c)/(a+b) | EBGM05>2 |
|  | 95%CI=e^ln(EBGM)±1.96(1/a+1/b+1/c+1/d)^0.5^ |  |

Equation: a, number of reports containing both the target drug and target adverse drug reactions; b, number of reports containing other drug adverse reactions that contain the targeted drug; c, number of reports containing the target adverse drug reaction of other drugs; d, number of reports containing other drugs and other adverse drug reactions. 95%CI, 95% confidence interval; N, the number of reports; χ^2^, chi-squared; IC, information component; IC025, the lower limit of 95% CI of the IC; E(IC), the IC expectations; V(IC), the variance of IC; EBGM, empirical Bayesian geometric mean; EBGM05, the lower limit of 95% CI of EBGM.

**Supplementary Table S3** The frequent adverse events for ifosfamide excluding common medication co-usage at the PT level from FAERS data

| PT | n | ROR(95%CI) | PRR(χ^2^) | EBGM(EBGM05) | IC(IC025) |
| --- | --- | --- | --- | --- | --- |
| Encephalopathy | 93 | 117.26 ( 95.2 - 144.44 ) | 112.03 ( 10193.93 ) | 111.55 ( 93.7 ) | 6.8 ( 6.5 ) |
| Confusional state | 64 | 11.75 ( 9.16 - 15.07 ) | 11.42 ( 609.57 ) | 11.41 ( 9.27 ) | 3.51 ( 3.15 ) |
| Neurotoxicity | 55 | 100.06 ( 76.51 - 130.86 ) | 97.42 ( 5230.61 ) | 97.06 ( 77.54 ) | 6.6 ( 6.21 ) |
| Disorientation | 27 | 19.58 ( 13.39 - 28.62 ) | 19.33 ( 469.39 ) | 19.32 ( 14.06 ) | 4.27 ( 3.72 ) |
| Agitation | 25 | 9.8 ( 6.61 - 14.54 ) | 9.69 ( 195.07 ) | 9.69 ( 6.97 ) | 3.28 ( 2.71 ) |
| Somnolence | 22 | 3.21 ( 2.11 - 4.88 ) | 3.18 ( 33.06 ) | 3.18 ( 2.24 ) | 1.67 ( 1.07 ) |
| Mental status changes | 21 | 22.09 ( 14.37 - 33.97 ) | 21.88 ( 418.27 ) | 21.86 ( 15.25 ) | 4.45 ( 3.83 ) |
| Tremor | 18 | 3.13 ( 1.97 - 4.98 ) | 3.11 ( 25.87 ) | 3.11 ( 2.11 ) | 1.64 ( 0.97 ) |
| Hallucination | 18 | 7.15 ( 4.49 - 11.37 ) | 7.09 ( 94.32 ) | 7.09 ( 4.81 ) | 2.83 ( 2.16 ) |
| Myoclonus | 15 | 36.96 ( 22.23 - 61.44 ) | 36.7 ( 520.25 ) | 36.65 ( 23.95 ) | 5.2 ( 4.47 ) |
| Lethargy | 13 | 6.54 ( 3.79 - 11.29 ) | 6.51 ( 60.64 ) | 6.51 ( 4.12 ) | 2.7 ( 1.93 ) |
| Toxic encephalopathy | 11 | 68.98 ( 38.11 - 124.84 ) | 68.61 ( 731.06 ) | 68.44 ( 41.66 ) | 6.1 ( 5.26 ) |
| Unresponsive to stimuli | 10 | 11.5 ( 6.18 - 21.4 ) | 11.44 ( 95.32 ) | 11.44 ( 6.8 ) | 3.52 ( 2.64 ) |
| Abnormal behaviour | 9 | 6.33 ( 3.29 - 12.19 ) | 6.31 ( 40.22 ) | 6.31 ( 3.65 ) | 2.66 ( 1.74 ) |
| Delirium | 8 | 6.89 ( 3.44 - 13.79 ) | 6.87 ( 40.1 ) | 6.86 ( 3.84 ) | 2.78 ( 1.81 ) |
| Coma | 8 | 4.87 ( 2.43 - 9.74 ) | 4.85 ( 24.47 ) | 4.85 ( 2.71 ) | 2.28 ( 1.31 ) |
| Restlessness | 6 | 4.72 ( 2.12 - 10.52 ) | 4.71 ( 17.54 ) | 4.71 ( 2.41 ) | 2.24 ( 1.14 ) |
| Altered state of consciousness | 6 | 8.39 ( 3.77 - 18.71 ) | 8.37 ( 38.95 ) | 8.37 ( 4.28 ) | 3.07 ( 1.97 ) |
| Extrapyramidal disorder | 6 | 6.62 ( 2.97 - 14.75 ) | 6.6 ( 28.51 ) | 6.6 ( 3.37 ) | 2.72 ( 1.63 ) |
| Psychotic disorder | 6 | 5.89 ( 2.64 - 13.14 ) | 5.88 ( 24.31 ) | 5.88 ( 3.01 ) | 2.56 ( 1.46 ) |
| Status epilepticus | 5 | 13.08 ( 5.44 - 31.47 ) | 13.05 ( 55.62 ) | 13.05 ( 6.26 ) | 3.71 ( 2.53 ) |
| Hallucination, visual | 5 | 7.18 ( 2.98 - 17.26 ) | 7.16 ( 26.51 ) | 7.16 ( 3.44 ) | 2.84 ( 1.66 ) |
| Incoherent | 5 | 21.85 ( 9.08 - 52.58 ) | 21.8 ( 99.17 ) | 21.79 ( 10.45 ) | 4.45 ( 3.27 ) |
| Psychomotor hyperactivity | 5 | 8.5 ( 3.53 - 20.45 ) | 8.48 ( 33.01 ) | 8.48 ( 4.07 ) | 3.08 ( 1.9 ) |
| Sopor | 4 | 8.28 ( 3.1 - 22.08 ) | 8.26 ( 25.53 ) | 8.26 ( 3.63 ) | 3.05 ( 1.75 ) |
| Gaze palsy* | 3 | 51.75 ( 16.66 - 160.76 ) | 51.67 ( 148.79 ) | 51.58 ( 19.98 ) | 5.69 ( 4.24 ) |
| Motor dysfunction | 3 | 8.7 ( 2.8 - 26.99 ) | 8.68 ( 20.4 ) | 8.68 ( 3.37 ) | 3.12 ( 1.67 ) |
| Coordination abnormal | 3 | 6.03 ( 1.94 - 18.71 ) | 6.02 ( 12.56 ) | 6.02 ( 2.33 ) | 2.59 ( 1.15 ) |
| Tonic clonic movements | 3 | 67.44 ( 21.7 - 209.57 ) | 67.34 ( 195.57 ) | 67.17 ( 26.01 ) | 6.07 ( 4.62 ) |
| Brain oedema* | 3 | 6.97 ( 2.24 - 21.62 ) | 6.96 ( 15.3 ) | 6.96 ( 2.7 ) | 2.8 ( 1.35 ) |
| Clumsiness | 3 | 31.06 ( 10 - 96.46 ) | 31.02 ( 87.06 ) | 30.99 ( 12.01 ) | 4.95 ( 3.51 ) |

*:Not mentioned in the bevacizumab instruction. Abbreviations: CI, confidence interval; ROR, reporting odds ratio; PRR, proportional reporting ratio; EBGM, empirical Bayesian geometric mean; EBGM05, the lower limit of the 95% confidence interval of EBGM; IC, information component; IC025, the lower limit of the 95% confidence interval of the IC.

**Supplementary Table S4** Cases of ifosfamide-related Central Neurotoxicity in the literature

| **References** | **Age** | **Gender** | **Indication** | **Ifosfamide Regimen** | **Cycle** | **Central Neurotoxicity Events** | **Treatment** | **Outcome** | **Recovery time** |
| --- | --- | --- | --- | --- | --- | --- | --- | --- | --- |
| Yu Furui et al.^(1)^ | 3 | Female | Ewing's sarcoma | 1.8g/m^2^ d1-5 | 5 | Impaired consciousness, bilateral eye deviation, limb weakness | Midazolam, methylene blue | Recovery | N/A |
| Daniel Taupin et al.^(2)^ | 60 | Female | Epithelioid carcinosarcoma | 2.5 g/m^2^ d1-5 | 1 | Lethargic, confused, eyelid fluttering, myoclonic facial | Methylene blue, thiamine, levetiracetam | Fatal | - |
| J. L. Alonso, et al.^(3)^ | 46 | Female | Ovarian adenocarcinoa | 2g/m^2^ d1, 1.5g/m^2^ d2-3 | 1 | Lethargy, alertness, stupor, echolalia, delirium | Lorazepam, methylene blue | Recovery | 5 hours |
| B. Kasper et al.^(4)^ | 30 | Female | Ewing sarcoma | 4g/m^2^ d1-3 | N/A | Encephalopathy | N/A | Recovery | N/A |
| Joseph R.DiMaggio, M.D. et al.^(5)^ | 59 | Female | Ovarian adenocarcinoa | 1.71 g/m^2^ d1-6 | 3 | Visual and auditory phenomena | N/A | Recovery | 2 days |
|  | 44 | Female | Breast cancer | 2.85 g/m^2^ d1-6 | N/A | Visual hallucinations | Haloperidol | Recovery | 2 days |
|  | 47 | Female | Breast cancer | 1.71 g/m^2^ d1-3 | 2 | Auditory hallucinations | Droperidol | Recovery | 2 days |
|  | 50 | Male | Non-Hodgkin's lymphoma | 2.4 g/m^2^ d1-6 | N/A | Visual hallucinations | Haloperidol | Fatal | - |
|  | 55 | Female | Breast cancer | 3.3 g/m^2^ d1-6 | N/A | Visual and auditory hallucinations | Droperidol | Recovery | N/A |
| You-Jung Shin et al.^(6)^ | 54 | Female | Ovarian cancer | 1.2 g/m^2^ d1-3 | 1 | Stupor, disorientation, stereotyped movements, emotional instability | N/A | Fatal | - |
|  | 46 | Female | Ovarian cancer | 1.2 g/m^2^ d1-5 | 1 | Delirium, disorientation, visual and auditory hallucinations, stereotyped movements, emotional instability | N/A | Fatal | - |
| Turner, A R et al.^(7)^ | 47 | Male | T-cell non-Hodgkin’s lymphoma | 5 g/m^2^ d1-5 | 1 | Encephalopathy | Methylene blue | Recovery | 2 days |
|  | 38 | Male | Embryonal sarcoma | 1.8 g/m^2^ d1-5 | 1 | Encephalopathy | Methylene blue | Recovery | 1 day |
|  | 27 | Male | Synovial sarcoma | 1.8 g/m^2^ d1-5 | 2 | Encephalopathy | Methylene blue | Recovery | 1 day |
| C. Kerdudo et al.^(8)^ | 16 | Female | Synovialosarcom | 3g/m^2^ d1-2 | 3 | Visual hallucinations, logorrhea, psychomotor agitation, insomnia | Clorazepate, methylene blue | Recovery | 2 days |
| Ataseven, Eda et al.^(9)^ | 14 | Female | Bone tumor, | 1.8 g/m^2^ d1-5 | 2 | Generalized tonic-clonic seizure | Diazepam | Recovery | few minutes |
|  | 9 | Female | Osteosarcoma | 2.8g/m^2^ d1-5 | 1 |  | Midazolam, levatiracetam | Recovery | N/A |
|  | 11 | Female | Medulloblastoma | 1.8 g/m^2^ d1-5 | 1 | Poorly responsive , somnolence, coma | Methylene blue, thiamine | Recovery | 2 days |
| Maria A. et al.^(10)^ | 4 | Male | Wilms tumor | 1.8 g/m^2^ d1-5 | 2 | Comatose, seizure | Diphenhydramine, naloxone | Recovery | 5 days |
|  | 4.5 | Female | Neuroblastoma | 1.8 g/m^2^ d1-5 | 2 | Comatose, seizure | Phenytoin | Recovery | 7 days |
| Norwood R. et al.^(11)^ | 61 | Male | Adenocarcinoma of the lung and a mixed large cell-lymphocytic lymphoma. | 1 g/m^2^ d1-5 | 1 | Confused, agitated, myoclonus, muscular spasticity, mute | Diphenhydramine | Recovery | 3 days |
| Lisa J. Miller et al.^(12)^ | 56 | Male | Folicularmixed cell non-Hodgkin's lymphoma | 1.33 g/m^2^ d1-3 | 1 | Drowsiness, confusion, disorientation, tremors in the hands feet, blank staring, meaningless speech, imitative movements, confusion | Haloperidol | Recovery | 3 days |
| Nancy A. Simonian, MD et al.^(13)^ | 59 | Female | Ovarian adenocarcinoa | 1 g/m^2^ d1-3 | N/A | Poorly responsive, mute | Diazepam. | Recovery | 4 hours |
|  | 32 | Female |  | 1.5 g/m^2^ d1-3 | N/A | Catatonic |  | Recovery | 4 minutes |
| Jennette I. McVay et al.^(14)^ | 43 | Male | Follicular and diffuse large cell lymphoma | 1.33g/m^2^ d1-3 | 1 | Restless, confused, irritable, disoriented | Lorazepam, haloperidol | Recovery | N/A |
| Alessandro Comandone et al.^(15)^ | 24 | Male | Osteosarcoma | 3.75 g/m^2^ d1-4 | 4 | Visual and auditory hallucinations, memory and orientation disturbances | Haloperidol | Recovery | 2 days |
|  |  |  |  | 1 g/m^2^ d1-14 | 1 | Confused, agitated, seizure | Diazepam |  | 2 days |
| Thomas Meyer et al .^(16)^ | 51 | Male | Plasmacytoma | 2g/m^2^ d1-2 | N/A | Asterixis | N/A | Recovery | 1 day |
| Antonella Brunello et al.^(17)^ | 66 | Male | Small- cell lung cancer | 3g/m^2^ d1 | 3 | Disorientation,visual and auditory hallucinations, stereotyped movements, emotional instability | Methylene blue | Fatal | - |
|  | 76 | Female | Uterine sarcoma | 1.8g/m^2^ d1-5 | 1 | Agitation, confusion | - | Recovery | 7 days |
|  | 77 | Female | Sarcoma | 1g/m^2^ d1-5 | 1 | Depression, delirium, agitation | Methylene blue | Recovery | 1 day |
|  | 70 | Male | Multiple myeloma | 2.5g/m^2^ d1-3 | 1 | Mental confusion, disorientation | Methylene blue | Recovery | 2 days |
|  | 70 | Female | Soft-tissue sarcoma | 3g/m^2^ d1-3 | 1 | Depression, prolonged crying, hallucinations, cognitive impairment, miosis | Methylene blue | Recovery | 3 days |
| Bethany Ames et al.^(18)^ | 16 | Male | Osteosarcoma | 2.8g/m^2^ d1-5 | 2 | Somnolent, confusion, head shaking, facial twitching, hand tremors ,hemiballismicarm, leg movements | Methylene blue | Recovery | 2 days |
|  | 7 | Female | Osteosarcoma | 2.8g/m^2^ d1-5 | 1 | Agitation, hallucinations, delirium, alertness | Methylene blue | Recovery | 1 day |
| Petros Giovanis et al.^(19)^ | 58 | Female | Sarcoma | 1g/m^2^ d1-5 | 1 | Lethargy, alertness, stupor, anxiety, agitation | Methylene blue | Recovery | 1 day |
| Sara Ramos Linares et al^.(20)^ | 38 | Male | Mixed-cellularity classical Hodgkin’s lymphoma | 2 g/m^2^ d1-4 | 1 | Confusional, agitation, anxiety, restlessness, dystonia, dysarthria, salivation, hypokinesia, tremor, mutism, lethargy , hallucinations | Benzodyazepines,  biperiden | Recovery | 3 weeks |
| Anusha Vakiti et al.^(21)^ | 47 | Male | Cutaneous T-cell lymphoma with large cell transformation | 5g/m^2^ d1 | 1 | Agitation, confusion,drowsiness, stupor | Methylene blue | Recovery | 2 days |
|  | 38 | Female | Mycosis fungoides with large cell transformation | 5g/m^2^ d1 | 1 | Lethargic, somnolent, confused, jerky  movements extremities, twitching of the right eye | Methylene blue | Recovery | 3 days |
| Hakan Sarbay et al.^(22)^ | 15 | Female | Osteosarcoma | 14g/m^2^ d1-3 | 1 | Generalized tonic clonic fits | Diazepam | Recovery | N/A |
| Gabriel Chain et al.^(23)^ | 11 | Female | Pineal tumor | 1.8g/m^2^ d1-5 | 4 | Drowsiness, confusion | Methylene blue | Recovery | 1 day |
| Ambika Menon et al.^(24)^ | 25 | Female | Round cell sarcoma | 1.8g/m^2^ d1-5 | 1 | Agitated, labile, defiant | Haloperidol, lorazepam, olanzapine, methylene blue , thiamine | Recovery | 4 days |
| Nalla Silva Baticam et al.^(25)^ | 15 | Male | Non-Germinomatus Germ Cell Tumors | N/A | 4 | Blurry vision, maculopathy | Methylprednisolone | Recovery | 13 days |
| Yeager J et al.^(26)^ | 21 | Male | Chondrosarcoma | N/A | 2 | Encephalopathy | Methylene blue,  thiamine | Recovery | 2 days |
| A Bhardwaj et al.^(27)^ | 71 | Male | Carcinosarcoma | 1.5g/m^2^ d1-5 | 1 | Nonconvulsive status epilepticus | Phenytoin | Recovery | 3 days |
| C. Kirch et al.^(28)^ | 18 | Female | Olfactory neuroblastoma | 2.4g d1-2 | N/A | Consciousness, epilepticus | Phenobarbital | Recovery | 2 days |
| Shuper, A et al.^(29)^ | 5 | N/A | Desmoplastic small round-cell tumor | 1.8g/m^2^ d1-5 | 4 | Encephalopathy | N/A | Fatal | - |
| Gonzalez-Angulo, Ana M et al.^(30)^ | 46 | Male | Bladder cancer | 1.5g/m^2^ d1-3 | 1 | Encephalopathy | Lorazepam, droperidol | Recovery | 3 days |
| Dietrich, Jörg et al.^(31)^ | 38 | Male | Testicular germ cell tumor | 2g/m^2^ d1-5 | 1 | Decreased alertness , depressive syndrome, severely impaired in mental function, orientation, and psychomotor activity and urinary incontinence | N/A | Recovery | 3-5 days |
| Park, I S et al.^(32)^ | 41 | Female | Cervical cancer | 1 g/m^2^ d1-5 | 1 | Affective dysfunction, seizure, urinary incontinence, agnosia, aphasia, ataxia | N/A | Recovery | N/A |
|  | 41 | Female | Cervical cancer | 1 g/m^2^ d1-5 | N/A | Mental change (stupor to semicoma) , generalized tonic–clonic seizure, affective and cognitive  dysfunction, dizziness, urinary incontinence, agnosia, aphasia, ataxia | Methylene blue | Recovery | 1 day |
| Alici-Evcimen, Yesne et al.^(33)^ | 44 | Female | Burkitt’s lymphoma | 1.5 g/m^2^ d1-5 | 2 | Agitation, grandiose delusions | Haloperidol, lorazepam, methylene blue | Recovery | 2 days |
| Hernández, Ricardo et al.^(34)^ | 67 | Male | Lung carcinoma | 3g/m^2^ d1 | 1 | Episodes of anxiety alternating with depression, visual hallucinations, anxiety, insomnia, unjustified fear and delusions of persecution | Haloperidol, risperidone | Fatal | - |
| Imtiaz, Saba et al.^(35)^ | 32 | Female | Diffuse large B cell lymphoma | N/A | 1 | Encephalopathy | Methylene blue, thiamine, methylprednisolone, phenytoin | Recovery | 3 days |
| Kataria, Pritam Sureshchandra et al.^(36)^ | 25 | Male | Philadelphia chromosome negative B-cell acute lymphoblastic  lymphoma | 4g/m^2^ d1-3 | 1 | Encephalopathy,seizure | Methylene blue | Recovery | 3 days |
| Gharaibeh, Eyad Z et al. ^(37)^ | 64 | Female | Diffuse large B-cell lymphoma | 2g/m^2^ d1-3 | 1 | Altered mental status, slurred speech, unsteady gait, myoclonic jerks, dysarthria | Methylene blue, thiamine | Recovery | 1 day |
| Hamilton, Juliette E et al.^(38)^ | 71 | Female | Pleomorphic sarcoma | N/A | 2 | Encephalopathy | Methylene blue, thiamine | Recovery | 10 minutes |
| Ali Mohamed, Daoud et al.^(39)^ | 13 | Male | Rhabdomyosarcoma | N/A | N/A | Seizures | N/A | Recovery | N/A |
| Primavera, Alberto et al.^(40)^ | 27 | Female | Non-Hodgkin’s lymphoma | 2.5 g/m^2^ d1-5 | 1 | Poorly responsive, somnolence, mute | Diazepam, methylene-blue | Recovery | 2 days |
|  | 57 | Female |  | 1.5 g/m^2^ d1-5 | 1 | Memory impairment, urinary incontinence, unresponsiveness to verbal command, absence of spontaneous speech, echolalia | Diazepam | Recovery | 2 days |
| Claessens, Arthur et al.^(41)^ | 58 | Female | Osteosarcoma | 3 g/m^2^ d1-3 | 1 | Confusion, aphasia, somnolence, agitation, aggressive behavior | Methylene blue | Recovery | 6 days |
| Pinilla Rello, Andrea et al.^(42)^ | 30 | Female | Soft tissue sarcoma | 2.5g/m^2^ d1-3 | 1 | Encephalopathy | Methylene-blue, thiamine, dexmedetomidine | Recovery | 3 days |

N/A: data not available

**References**

1.	Furui Y, Komori K, Kurata T, Sakashita K. Ifosfamide-Induced Encephalopathy Successfully Prevented by Methylene Blue: A Pediatric Case Report and Review of the Literature. Cureus. 2023;15(6):e40213.

2.	Taupin D, Racela R, Friedman D. Ifosfamide chemotherapy and nonconvulsive status epilepticus: case report and review of the literature. Clin EEG Neurosci. 2014;45(3):222-5.

3.	Alonso JL, Nieto Y, Lopez JA, Martin M, Diaz-Rubio E. Ifosfamide encephalopathy and methylene-blue: a case report. Ann Oncol. 1996;7(6):643-4.

4.	Kasper B, Harter C, Meissner J, Bellos F, Krasniqi F, Ho AD, et al. Prophylactic treatment of known ifosfamide-induced encephalopathy for chemotherapy with high-dose ifosfamide? Support Care Cancer. 2004;12(3):205-7.

5.	DiMaggio JR, Brown R, Baile WF, Schapira D. Hallucinations and ifosfamide-induced neurotoxicity. Cancer. 1994;73(5):1509-14.

6.	Shin YJ, Kim JY, Moon JW, You RM, Park JY, Nam JH. Fatal Ifosfamide-induced metabolic encephalopathy in patients with recurrent epithelial ovarian cancer: report of two cases. Cancer Res Treat. 2011;43(4):260-3.

7.	Turner AR, Duong CD, Good DJ. Methylene blue for the treatment and prophylaxis of ifosfamide-induced encephalopathy. Clin Oncol (R Coll Radiol). 2003;15(7):435-9.

8.	Kerdudo C, Orbach D, Sarradet JL, Doz F. Ifosfamide neurotoxicity: an atypical presentation with psychiatric manifestations. Pediatr Blood Cancer. 2006;47(1):100-2.

9.	Ataseven E, Goktepe SO, Kantar M. Ifosfamide-related encephalopathy with severe clinical presentations in children with cancer. J Oncol Pharm Pract. 2021;27(8):2018-22.

10.	Gieron MA, Barak LS, Estrada J. Severe encephalopathy associated with ifosfamide administration in two children with metastatic tumors. J Neurooncol. 1988;6(1):29-30.

11.	Anderson NR, Tandon DS. Ifosfamide extrapyramidal neurotoxicity. Cancer. 1991;68(1):72-5.

12.	Miller LJ, Eaton VE. Ifosfamide-induced neurotoxicity: a case report and review of the literature. Ann Pharmacother. 1992;26(2):183-7.

13.	Simonian NA GF, Chiappa KH. . Ifosfamide causes a diazepam-sensitive encephalopathy. Neurology. 1993 43(12):2700-2.

14.	McVay JI, Wood AM. Suspected ifosfamide-induced neurotoxicity. Pharmacotherapy. 1999;19(12):1450-5.

15.	Comandone A LW, Picci P, et al. Two episodes of ifosfamide-related neurotoxicity in the same patient following different schedules and doses of the drug. Tumori 2000;86(6):483-6.

16.	Meyer T, Ludolph AC, Munch C. Ifosfamide encephalopathy presenting with asterixis. J Neurol Sci. 2002;199(1-2):85-8.

17.	Brunello A BU, Rossi E, et al. . Ifosfamide-related encephalopathy in elderly patients : report of five cases and review of the literature. . Drugs Aging. 2007;24(11):967-73.

18.	Ames B, Lewis LD, Chaffee S, Kim J, Morse R. Ifosfamide-induced encephalopathy and movement disorder. Pediatr Blood Cancer. 2010;54(4):624-6.

19.	Giovanis P, Garna A, Marcante M, Nardi K, Giusto M. Ifosfamide encephalopathy and use of methylene blue. A case report of different sequential neurotoxicity. Tumori. 2009;95(4):545-6.

20.	Ramos Linares S, Brena Atienza J, Chafer Rudilla M, Rios Rull P, Cabello Rodriguez A, Merino Alonso J. Severe ifosfamide-induced neurotoxicity: a case report. Pharm World Sci. 2010;32(2):109-11.

21.	Vakiti A, Pilla R, Alhaj Moustafa M, Joseph JJ, Shenoy AG. Ifosfamide-Induced Metabolic Encephalopathy in 2 Patients With Cutaneous T-Cell Lymphoma Successfully Treated With Methylene Blue. J Investig Med High Impact Case Rep. 2018;6:2324709618786769.

22.	Sarbay H, Demir UF, Yilmaz G, Atay AA, Malbora B. Ifosfamide induced encephalopathy in a child with osteosarcoma. J Oncol Pharm Pract. 2021;27(5):1302-6.

23.	Chain G, Kalia M, Kestenbaum K, Pappas L, Sechser-Perl A, Campino GA, et al. A novel case of prolonged Ifosfamide encephalopathy and long-term treatment with methylene blue: a case report and review of literature. BMC Pediatr. 2022;22(1):76.

24.	Menon A, Enunwa CA, Read WL, James KP. Rare Delayed Ifosfamide Encephalopathy: A Case Report of Chemotherapeutic Neurotoxicity. Case Rep Oncol. 2024;17(1):202-7.

25.	Silva Baticam N, Roberge X, Laliberte I, Gauquelin L, Liu A, Hawkins C, et al. Ifosfamide-Related Optic Nerve Toxicity: Case Description and Review of the Literature. Pediatr Blood Cancer. 2025;72(8):e31799.

26.	Yeager J, Basnet A. Delayed Ifosfamide Neurotoxicity. Am J Ther. 2020;27(6):e613-e4.

27.	Bhardwaj A, Badesha PS. Ifosfamide-induced nonconvulsive status epilepticus. Ann Pharmacother. 1995;29(12):1237-9.

28.	Kirch C, Gachot B, Germann N, Blot F, Nitenberg G. Recurrent ifosfamide-induced hyponatraemia. Eur J Cancer. 1997;33(14):2438-9.

29.	Shuper A, Stein J, Goshen J, Kornreich L, Yaniv I, Cohen IJ, et al. Subacute Central Nervous System Degeneration in a Child: An Unusual Manifestation of Ifosfamide Intoxication. Journal of Child Neurology. 2000;15(7):481-3.

30.	Gonzalez-Angulo AM, Orzano JA, Davila E. Ifosfamide-induced encephalopathy. South Med J. 2002;95(10):1215-7.

31.	Dietrich J, Marienhagen J, Schalke B, Bogdahn U, Schlachetzki F. Vascular neurotoxicity following chemotherapy with cisplatin, ifosfamide, and etoposide. Ann Pharmacother. 2004;38(2):242-6.

32.	Park IS, Lee HJ, Lee YS, Hwang JS, Lee MS. Ifosfamide-induced encephalopathy with or without using methylene blue. Int J Gynecol Cancer. 2005;15(5):807-10.

33.	Alici-Evcimen Y, Breitbart WS. Ifosfamide neuropsychiatric toxicity in patients with cancer. Psychooncology. 2007;16(10):956-60.

34.	Hernandez R, Juan O, Alberola V. Ifosfamide-induced psychosis. Acta Oncol. 2004;43(1):119-20.

35.	Saba Imtiaz NM. Ifosfamide neurotoxicty in a young female with a remarkable response to thiamine. J Pak Med Assoc. 2010;60(10):867-9.

36.	Kataria PS, Kendre PP, Patel AA. Ifosfamide-induced Encephalopathy Precipitated by Aprepitant: A Rarely Manifested Side Effect of Drug Interaction. J Pharmacol Pharmacother. 2017;8(1):38-40.

37.	Gharaibeh EZ, Telfah M, Powers BC, Salacz ME. Hydration, methylene blue, and thiamine as a prevention regimen for ifosfamide-induced encephalopathy. J Oncol Pharm Pract. 2019;25(7):1784-6.

38.	Hamilton JE, Alexander M, Kelleher FC. Ifosfamide-induced encephalopathy: the EEG with frontal intermittent delta activity, and rapid resolution with methylene blue: A case report. Clin Sarcoma Res. 2020;10(1):25.

39.	Ali Mohamed D, Semedo A, Adeyemi B, Hessissen L, El Kababri M, Allali N, et al. Reversible Encepahlopathy Induced by Ifosfamide with Brain Imaging. Glob Pediatr Health. 2021;8:2333794X211030415.

40.	Alberto Primavera DA, Leonardo Cocito. Ifosfamide encephalopathy and nonconvulsive status epilepticus. THE CANADIAN JOURNAL OF NEUROLOGICAL SCIENCES. 2002;29(2):4.

41.	Claessens A, Manchart A, Boufraine M, Guignard A, Bergeot A, Kieffer A, et al. Neuron-Specific Enolase as a Biomarker in Ifosfamide-Induced Encephalopathy: A Case Report. Case Rep Oncol. 2025;18(1):800-8.

42.	Pinilla Rello A, Huarte Lacunza R, Tejada Artigas A. Ifosfamide-induced encephalopathy and concomitant use of netupitant/palonosetron in a patient with soft tissue sarcoma: a case report. Eur J Hosp Pharm. 2025.

**Supplementary Table S5** JBI Quality assessment Matrix of IFO cases

| ***Study*** | **Year** | **Design** | **N** | **Q1**  **Demo graphics clearly described** | **Q2 History presented as a time line** | **Q3 Current condition clearly described** | **Q4 Diagnostic tests & results described** | **Q5 Intervention clearly described** | **Q6 Post-intervention condition described** | **Q7 Adverse events identified & described** | **Q8 Take away lessons provided** | **Yes/8** |
| --- | --- | --- | --- | --- | --- | --- | --- | --- | --- | --- | --- | --- |
| Furui Y | 2023 | case report | 1 | Y | Y | Y | Y | Y | Y | Y | Y | 8 |
| Taupin D | 2014 | case report | 1 | Y | Y | Y | Y | Y | Y | Y | Y | 8 |
| Alonso JL | 1996 | case report | 1 | Y | Y | Y | Y | Y | Y | Y | Y | 8 |
| Kasper B | 2004 | case report | 1 | Y | Y | **U** | **U** | Y | Y | Y | Y | 6 |
| DiMaggio JR | 1994 | case series | 6 | Y | Y | Y | Y | Y | Y | Y | Y | 8 |
| Shin YJ | 2011 | case series | 2 | Y | Y | Y | Y | Y | Y | Y | Y | 8 |
| Turner AR | 2003 | case series | 4 | Y | Y | Y | Y | Y | Y | Y | Y | 8 |
| Kerdudo C | 2006 | case report | 1 | Y | Y | Y | Y | Y | Y | Y | Y | 8 |
| Ataseven E | 2021 | case series | 3 | Y | Y | Y | Y | Y | Y | Y | Y | 8 |
| Gieron MA | 1988 | case series | 2 | Y | Y | Y | Y | Y | Y | Y | Y | 8 |
| Anderson NR | 1991 | case report | 1 | Y | Y | Y | Y | Y | Y | Y | Y | 8 |
| Miller LJ | 1992 | case report | 1 | Y | Y | Y | Y | Y | Y | Y | Y | 8 |
| Simonian NA | 1993 | case series | 2 | Y | Y | Y | Y | Y | Y | Y | Y | 8 |
| McVay JI | 1999 | case report | 1 | Y | Y | Y | Y | Y | Y | Y | Y | 8 |
| Comandone A | 2000 | case report | 1 | Y | Y | Y | Y | Y | Y | Y | Y | 8 |
| Meyer T | 2002 | case report | 1 | Y | Y | Y | Y | Y | Y | Y | Y | 8 |
| Brunello A | 2007 | case series | 5 | Y | Y | Y | Y | Y | Y | Y | Y | 8 |
| Ames B | 2010 | case series | 2 | Y | Y | Y | Y | Y | Y | Y | Y | 8 |
| Giovanis P | 2009 | case report | 1 | Y | Y | Y | Y | Y | Y | Y | Y | 8 |
| Ramos Linares S | 2010 | case report | 1 | Y | Y | Y | Y | Y | Y | Y | Y | 8 |
| Vakiti A | 2018 | case series | 2 | Y | Y | Y | Y | Y | Y | Y | Y | 8 |
| Sarbay H | 2020 | case report | 1 | Y | Y | Y | Y | Y | Y | Y | Y | 8 |
| Chain G | 2022 | case report | 1 | Y | Y | Y | Y | Y | Y | Y | Y | 8 |
| Menon A | 2024 | case report | 1 | Y | Y | Y | Y | Y | Y | Y | Y | 8 |
| Silva Baticam N | 2025 | case report | 1 | Y | Y | Y | Y | Y | Y | Y | Y | 8 |
| Yeager J | 2020 | case report | 1 | Y | Y | Y | Y | **U** | Y | Y | Y | 7 |
| Bhardwaj A | 1995 | case report | 1 | Y | Y | Y | Y | Y | Y | Y | Y | 8 |
| Kirch C | 1997 | case report | 1 | Y | Y | Y | Y | Y | Y | Y | Y | 8 |
| Shuper A | 2000 | case report | 1 | Y | Y | Y | Y | Y | Y | Y | Y | 8 |
| Gonzalez-Angulo AM | 2002 | case report | 1 | Y | Y | Y | Y | Y | Y | Y | Y | 8 |
| Dietrich J | 2004 | case report | 1 | Y | Y | Y | Y | Y | Y | Y | Y | 8 |
| Park IS | 2005 | case series | 2 | Y | Y | Y | Y | Y | Y | Y | Y | 8 |
| Alici-Evcimen Y | 2007 | case report | 1 | Y | Y | Y | Y | Y | Y | Y | Y | 8 |
| Hernandez R | 2004 | case report | 1 | Y | Y | Y | Y | Y | Y | Y | Y | 8 |
| Imtiaz S | 2010 | case report | 1 | Y | Y | Y | Y | Y | Y | Y | Y | 8 |
| Kataria PS | 2017 | case report | 1 | Y | Y | Y | Y | Y | Y | Y | Y | 8 |
| Gharaibeh EZ | 2018 | case report | 1 | Y | Y | Y | Y | Y | Y | Y | Y | 8 |
| Hamilton JE | 2020 | case report | 1 | Y | Y | Y | Y | **U** | Y | Y | Y | 7 |
| Mohamed DA | 2021 | case report | 1 | Y | Y | Y | Y | Y | Y | Y | Y | 8 |
| Primavera A | 2002 | case series | 2 | Y | Y | Y | Y | Y | Y | Y | Y | 8 |
| Claessens A | 2025 | case report | 1 | Y | Y | Y | Y | Y | Y | Y | Y | 8 |
| Pinilla Rello A | 2025 | case report | 1 | Y | Y | Y | **U** | Y | Y | Y | Y | 7 |

JBI = Joanna Briggs Institute. Quality appraisal followed the JBI Critical Appraisal Checklist for Case; Y = yes; U = unclear; N = no; NA = not applicable.

**Supplementary Table S6.** Treatments reported in 61 patients with ifosfamide-related central neurotoxicity

| **Treatment** | **Patients, n** | **Proportion, %** |
| --- | --- | --- |
| **Methylene blue** | **29** | **47.54** |
| **Benzodiazepines** | **16** | **26.23** |
| Diazepam | 7 | 11.48 |
| Lorazepam | 5 | 8.20 |
| Midazolam | 2 | 3.28 |
| Clorazepate | 1 | 1.64 |
| Unspecified benzodiazepines | 1 | 1.64 |
| **Antipsychotics** | **11** | **18.03** |
| Haloperidol | 8 | 13.11 |
| Droperidol | 3 | 4.92 |
| Olanzapine | 1 | 1.64 |
| Risperidone | 1 | 1.64 |
| **Thiamine** | **8** | **13.11** |
| **Phenytoin** | 3 | 4.92 |
| **Levetiracetam** | 2 | 3.28 |
| **Diphenhydramine** | 2 | 3.28 |
| **Methylprednisolone** | 2 | 3.28 |
| **Phenobarbital** | 1 | 1.64 |
| **Biperiden** | 1 | 1.64 |
| **Naloxone** | 1 | 1.64 |
| **Dexmedetomidine** | 1 | 1.64 |

Note: Some patients received combination therapy; therefore, percentages are not mutually exclusive.

Antipsychotics were counted by unique patients (n=11).
